# GsMTx-4 combined with exercise improves skeletal muscle structure and motor function in rats with spinal cord injury

**DOI:** 10.1371/journal.pone.0317683

**Published:** 2025-01-22

**Authors:** Xin Zhang, Xinyu Liu, Qianxi Li, Chenyu Li, Xinyan Li, Jinghua Qian, Jianjun Li, Xuemei Li

**Affiliations:** 1 School of Sport Medicine and Rehabilitation, Beijing Sport University, Beijing, China; 2 School of Rehabilitation Medicine, Capital Medical University, Beijing, China; 3 Department of Spinal and Neural Function Reconstruction, China Rehabilitation Research Center, Beijing, China; 4 Center of Neural Injury and Repair, Beijing Institute for Brain Disorders, Beijing, China; 5 China Rehabilitation Science Institute, Beijing, China; 6 Beijing Key Laboratory of Neural Injury and Rehabilitation, Beijing, China; Lorestan University, ISLAMIC REPUBLIC OF IRAN

## Abstract

Motor dysfunction and muscle atrophy are typical symptoms of patients with spinal cord injury (SCI). Exercise training is a conventional physical therapy after SCI, but exercise intervention alone may have limited efficacy in reducing secondary injury and promoting nerve regeneration and functional remodeling. Our previous research found that intramedullary pressure after SCI is one of the key factors affecting functional prognosis. It has been reported that GsMTx-4, a specific blocker of the mechanosensitive ion channels Piezo1, can protect the integrity of the neuromuscular junction and promote nerve regeneration, and thus has the potential as a therapeutic agent for SCI. In this study, we observed the combined and separate therapeutic effect of GsMTx-4 and exercise on the structure of the soleus muscle and motor function in rats with SCI. At 42 days post-injury, compared with SCI rats, the Basso–Beattie–Bresnahan score (P = 0.0007) and Gait Symmetry (P = 0.0002) were significantly improved after combination therapy. On histology of rat soleus muscle, compared with SCI rats, the combined treatment significantly increased the wet weight ratio, muscle fiber cross-sectional area and acetylcholinesterase (all P<0.0001). On histology of rat spinal tissue, compared with SCI rats, the combined treatment significantly increased neuron counts and BDNF levels, and significantly reduced the percentage of TUNEL-positive cells (all P<0.0001). On physiology of rat soleus muscle, compared with SCI rats, the combined treatment increased the succinate dehydrogenase expression (P<0.0001), while the expression of α-glycerophosphate dehydrogenase (P<0.0001) and GDF8 protein (P = 0.0008) decreased. Results indicate the combination therapy effectively improves histopathology of spinal cord and soleus muscle in SCI rats, enhancing motor function. This study was conducted on animal models, it offers insights for SCI treatment, advancing understanding of lower limb muscle pathology post-SCI. Further research is needed for clinical validation in the future.

## Introduction

Recovery of motor function in patients with SCI is the most urgent rehabilitation goal. Physical therapy methods such as exercise training are often used clinically to improve spinal cord function by enhancing, compensating, and replacing the remaining functions of nerves and muscles [1]. Previous studies have shown that body weight support treadmill training (BWSTT) may directly strengthen paralyzed muscles, relieve muscle atrophy caused by SCI to a certain extent, and improve the motor function of SCI patients [2, 3]. However, exercise training alone may have limited effects on promoting neural remodeling, improving the spinal microenvironment, neuroprotection, and reducing secondary injury after SCI [4–6]. Meanwhile, there may be a higher requirement for exercise intensity and the remaining neurological and functional characteristics of SCI patients [7–9]. Therefore, motor function recovery after SCI may require a practical and comprehensive treatment program, rather than mono or local therapy, to promote the functional network.

In our previous research, we found that spinal cord hemorrhage and edema rapidly occur in acute SCI [10]. Moreover, the resulting increase in intramedullary pressure may be essential for exacerbating secondary injury and hindering nerve repair [10, 11], which may be closely related to poor neurological prognosis in SCI patients [12]. Mechanical gated Piezo channels are the first class of mechanically gated non-selective cation channels found in mammals [13]. Previous studies have found through single-cell RNA sequencing analysis that the level of Piezo1 was significantly upregulated after SCI. This upregulation might be an adaptive response of cells to mechanical stress after SCI, regulating cell functions through the mechanically sensitive properties of Piezo1 [14]. The transmembrane structure of Piezo1 has non-selective cation permeability and can respond to mechanical stress stimuli on the cell membrane, influencing the ionic balance inside and outside the cell, such as causing the influx of Ca^2+^ into the cell [15]. Changes in the concentration of Ca^2+^ have a significant impact on the excitability of neurons and cell survival [16]. Calcium overload can activate calpain, affecting the normal binding and signal transduction between brain-derived neurotrophic factor (BDNF) and the TrkB receptor, thereby influencing the role of BDNF in aspects such as neuronal growth and synaptic plasticity [17]. Piezo1 channel may be related to the pathogenesis of SCI [18]. Down-regulation of Piezo1 may be beneficial in inhibiting inflammation at the injury site and improving the motor function of SCI mice [19]. GsMTx4 is a specific blocker of endogenous mechanosensitive ion channels, which can effectively block Piezo1 channels and reduce the influx of Ca^2+^ [20]. Using GsMTx4 to block Piezo1 channels shows neuroprotective and regenerative effects, such as stimulating nerve axon growth, promoting myelination, and reducing demyelination and neuronal damage in the central nervous system [21, 22]. These findings suggest that GsMTx4 may have potential as a therapeutic agent for SCI. In addition, GsMTx4 has been reported to regulate the response of skeletal muscle cells to mechanical stimuli, thereby influencing the structure and function of skeletal muscles. It shows certain application prospects in the treatment of skeletal muscle diseases [23, 24].

Some studies have reported that after SCI, exercise has multifaceted effects on skeletal muscle structure and muscle metabolism by regulating the activity changes of various markers, including promoting muscle cell growth and regulating energy metabolism pathways [25]. Long-term regular exercise can increase the content of succinate dehydrogenase (SDH) in skeletal muscles, thereby promoting cellular energy metabolism and mitochondrial function [26]. As a key enzyme in the tricarboxylic acid cycle, the activity of SDH can reflect the energy metabolism state of skeletal muscles [27]. Meanwhile, exercise can also increase the content and activity of glycerol-3-phosphate dehydrogenase (GPDH) in muscle tissues [28]. GPDH participates in the synthesis of glycerophospholipids and the process of gluconeogenesis, and it is crucial for maintaining the integrity of muscle cell membranes and energy supply [29]. In addition, long-term regular exercise can also keep the growth differentiation factor 8 (GDF8) at a relatively low level, which is helpful for muscle metabolism and function [30]. As a key factor in regulating muscle growth, changes in the level of GDF8 directly affect muscle mass and limit excessive muscle growth [31]. During the exercise process, with the frequent neuromuscular activities, the release and decomposition of acetylcholine at the neuromuscular junction are accelerated, and the activity of acetylcholinesterase (AChE) is correspondingly increased [32]. As a key enzyme in cholinergic synaptic transmission, the activity of AChE has an important impact on the signal transmission efficiency of the neuromuscular junction (NMJ) [33].

Based on these research findings, we hypothesized that GsMTx4 combined with BWSTT may help improve skeletal muscle structure, muscle metabolism and motor function in rats after SCI. The present study observed the combined and individual therapeutic effects of GsMTx4 and BWSTT on soleus muscle structure and motor function in rat SCI models. We aimed to find a systemic therapy that could simultaneously protect spinal cord neurons and block or alleviate rapid skeletal muscle atrophy to more effectively promote the recovery of motor function after SCI.

## Materials and methods

### Animals and grouping

Healthy female Sprague Dawley (SD) rats weighing 220-280g and aged 8–10 weeks were obtained from Beijing Vital River Laboratory Animal Technology Co., Ltd. (Beijing, China). All the animals were housed in an SPF environment with a temperature of 22 ± 1°C, a relative humidity of 50 ± 1%, and a light/dark cycle of 12/12 hr and had free access to food and water. All animal studies (including the rats euthanasia procedure) were done in compliance with the regulations and guidelines of Beijing Sport University institutional animal care and conducted according to the AAALAC and the IACUC guidelines.

**Fig 1:**
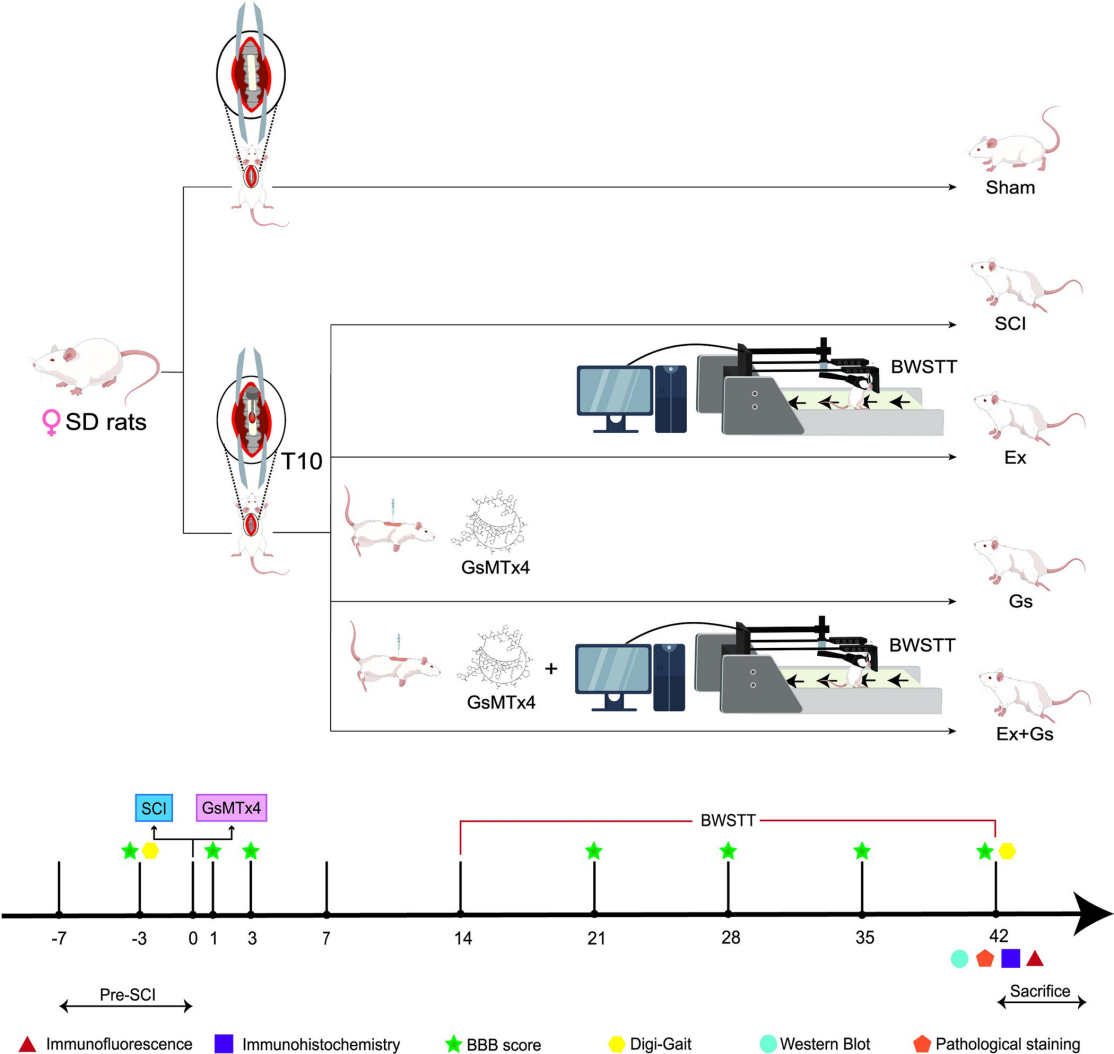
Grouping, experimental procedure, and time points. The rats were randomly divided into 5 groups, in which the SCI group, Ex group, Gs group, and Ex+Gs group underwent modeling surgery for spinal cord injury at the level of T10. GsMTx-4 was injected by a micro syringe after modeling; BBB score evaluation was performed on day 1 and 3 after modeling to determine whether the modeling was successful. BWSTT (body weight support treadmill training) started on day 14 after modeling in the Ex and Ex+Gs group rats. BBB score evaluation was performed every week for 4 consecutive weeks after training. At the end of the experiment, the digital footprint analysis system (Digital Gait) was used to evaluate the motor function of rats, and pathological staining, IHC, IF, and WB were performed. BBB: Basso–Beattie–Bresnahan; SCI: spinal cord injury; IHC: immunohistochemistry; IF: immunofluorescence; WB: Western Blot.

A total of 45 animals were used in this study, and the rats that met the standards were randomly divided into the following five groups using a random number table: Sham group, SCI group, Ex group, Gs group, and Ex+Gs group. Based on literature and laboratory experience, considering the feasibility of the experiment and the reliability of results, we have determined a sample size of 9 for each group. The grouping and experimental process are shown in **Fig 1**. In histology measures and digi-gait analysis, a blinded researcher performed the data collection and two different researchers did the analysis.

The Beijing Sport University ethics committee approved all experimental procedures, which were in accordance with the animal use and care guidelines. The study was conducted according to the ethical rules of the Animal Experiments and Experimental Animal Welfare Committee (2022033A).

### SCI model

Rats were subjected to gas anesthesia using 2% isoflurane (Shenzhen RWD Life Technology Co., Ltd., China). Rats in the Sham group only underwent laminectomy. IH Impactor (IH-0400, Precision Systems and Instrumentation IH Spinal Cord Impactor, USA) was used for the remaining four groups of rats to prepare the T10 SCI model, and the impact intensity was set to 200 kdynes. Preliminary criteria for successful modeling were tail swinging and vigorous twitching of the hind limbs. Rats that showed any movement of their lower limbs within 1 day of modeling were eliminated. After the model was successfully created, the rats were kept warm until they woke up and were returned to the corresponding cages. Fluid rehydration (1 mL of normal saline) and antibiotic (penicillin 100,000 U/per) were given as usual every day to prevent infection within 3 days after surgery. Assisted urination care was provided every 12 hours after surgery until spontaneous urination was restored.

### GsMTx-4 intervention

ddH_2_O was used to prepare GsMTx-4 (ab141871, Abcam, UK) stock solution at a concentration of 8 μM. A microsyringe pump (Quintessential, Stoelting, USA) was used for in situ injection into the spinal cord. A 10 μl microsyringe (Hamilton) was used, and a G33# needle was used to penetrate the dura mater vertically and inject separately rostral and caudal to the injury site (5 μl/animal; the injection speed was 1 μl/min). The Sham group was injected with the same dose of ddH_2_O.

### Exercise training

The rats in the Ex group and Ex+Gs group underwent BWSTT on the 14^th^ day after SCI modeling, and the rats were allowed to adapt to the BWSTT equipment before formal training. The rats from the Sham, SCI, and Gs groups rested in cages. This equipment was developed by a team from the Beijing Institute of Technology and has been successfully used in previous research [32]. The exercise speed was set to 5cm/s and weight loss to 80%-100% of body weight. Also, the degree of weight loss was gradually adjusted over time. The training was performed over a total of 4 weeks, 7 days a week, once a day, 20 minutes each time. In the first week of training, When the rat could not step independently, the experimenter would help the rat adapt to the bipedal walking mode on the running belt. After the rat adapted to the training equipment (step independently on their hind limbs), the degree of assistance was reduced. In order to ensure that the rat exert sufficient effort during BWSTT, the researchers monitored the entire training process and confirmed that the hind limb muscles of the rat actively contract to exert force and take steps forward to maximize the exercise of the hind limbs.

### Basso-Beattie-Bresnahan (BBB) score

The motor function of each group of rats was evaluated before the animal SCI model was established and on the 1, 3, 21, 28, 35, and 42 days after injury. The hindlimb movement of rats was divided into 22 levels according to the BBB score, where 0 points were assigned if there was no active movement of the hindlimb, and 21 points were assigned if the motor function was completely normal. This method is commonly used to evaluate the hindlimb movement ability of SCI rats [34]. Before the assessment, the rats were placed in an open field to adapt to the environment for 15 minutes. After adapting to the environment, they were observed for 5 minutes. Two researchers conducted the scoring using a blind approach; all assessors had received relevant training.

### Digi-gait analysis

The motor function of rats was evaluated using a rodent-specific digital footprint analysis system (Digi-Gait ^TM^, Mouse Specifics Inc., MA. USA). The treadmill speed was slowly increased to 5cm/s, a high-speed camera (Basler A602 camera, 150 fps, Basler AG, Ahrensburg, Germany) was used to record at least 5 consecutive gait cycles, three video recordings were made for each evaluation, and specialized analysis software (DigiGait ^TM^ analysis) was used to digitize the resulting images. The following 6 parameters were mainly evaluated: stance/swing ratio, stride frequency, paw area, hindlimb shared stance, gait symmetry, and forelimb weight support. The remaining parameters were measured on the rat hindlimb, except for gait symmetry and forelimb weight support. Hindlimb’s shared stance assesses the time the left and right hind paws are simultaneously in contact with the treadmill. Hindlimb’s shared stance may represent balance. Forelimb weight support = (width of each front paw + average touching width of front paw) / average width of the animal, and the width was calculated as the widest part of the body from the tip of the nose to the tail as the center axis. The stance/swing ratio is the time spent in the stance and swing phase. Stride frequency is the number of steps each paw takes per second (steps/s). The paw area is the maximum paw area at peak stance. Gait symmetry is the ratio of the forelimb step frequency to the hindlimb step frequency.

### Sample collection

The rats were anesthetized with 2% pentobarbital sodium. The spinal cord tissue about 5 mm from the center of the injured area was removed and placed in tissue fixative at 4°C overnight for subsequent experiments. The soleus muscle of the lower limb was surgically removed. After carefully removing the fat and excess connective tissue on the muscle surface, the wet weight of the muscle was measured, and the wet weight ratio = wet weight/body weight was calculated, after which it was placed in tissue fixative overnight at 4°C. Both ends of the muscle were trimmed and removed, the muscle belly was embedded in paraffin, and 4 μm-thick muscle transverse sections were cut for subsequent experiments. The remaining soleus muscle samples were quickly frozen in liquid nitrogen and stored at -80°C for subsequent testing.

### Muscle fiber count and cross-sectional area (CSA) measurement

The rat skeletal muscle cross-sectional specimens were serially paraffin sectioned, with a thickness of 4 μm. Hematoxylin and Eosin staining of the sections was performed according to the instructions for using the staining reagent, after which the morphological characteristics of skeletal muscle fibers were observed. The number and cross-sectional area of muscle fibers were measured using Image J (National Institutes of Health, Bethesda, MD, USA). On the cross-sectional image of each muscle, 5 squares not located at the edge of the image were randomly selected (making sure that all 4 of its borders were occupied by muscle fibers and excluding squares whose borders were not occupied by muscle fibers) and each selection was separately counted. The number of muscle fibers in the square was determined, and CSA was measured. When counting the number of muscle fibers in each square, the muscle fibers on the upper and left borders of the square were not counted.

### Nissl staining

Fixed spinal cord tissue was subjected to dehydration, dipping in wax, embedding, and sectioning at a thickness of 4 μm in preparation for Nissl staining (n = 6 per group). The Nissl-positive cells in the anterior horns of the spinal cord were counted (positive definition: intact cells, nuclei, and axons) in a double-blind manner, and the counts were averaged.

All stained images were scanned at high resolution with HistoFAXS 3.0 (Tissue Gnostics), and the pictures were viewed using the associated professional viewing software (FAXS viewer).

### TUNEL

TUNEL was used to detect apoptosis in anterior horn neurons of the spinal cord. In order to block endogenous peroxidase activity, sections were incubated with a methanol solution containing 0.2% H_2_O_2_ for 0.5 hours. Next, the sections were treated with a TUNEL reaction mixture (Millipore, Bedford, MA, USA), and the sections were kept in a 37°C incubator for 60 minutes. After HistoFAXS 3.0 was used to scan all tissue section images, the total number of cells in the anterior horns of the spinal cord and the number of TUNEL-positive cells were manually counted in a double-blind manner. The ratio was calculated as follows: TUNEL-positive cells% = (TUNEL-positive cells) / (total cells) × 100%.

### Immunohistochemistry (IHC)

After dewaxing the paraffin sections, they were placed in citric acid antigen recovery buffer (pH = 9.0) in a microwave oven for antigen recovery. Endogenous peroxidase blocking was done, and after blocking the sections with serum, they were incubated with primary antibodies (see **Table 1** for reagent usage) overnight at 4°C and then with secondary antibodies, after which they were developed with DAB (Diaminobenzidine) and counterstained with hematoxylin for nuclei. After HistoFAXS 3.0 was used to scan all tissue section images, the cross-sectional images were captured at 20x magnification and digitized using Image J. The mean OD (Optical Density) of GPDH from the images was automatically detected in a double-blind manner.

**Table 1:** Antibodies information.

Antibodies	Source	Identifier	Application
**Anti-Acetylcholinesterase**	Abcam, Cambridge, UK	ab183591	IF
**Anti-SDHA**	Abcam, Cambridge, UK	ab14715	IF
**Goat Anti-Rabbit (488)**	Servicebio, Wuhan, China	GB25303	IF
**Goat Anti-Mouse (CY3)**	Servicebio, Wuhan, China	GB21301	IF
**Anti-GPD1**	Arigo biolaboratories, Shanghai, China	ARG58861	IHC
**Goat Anti-Rabbit (HRP)**	Servicebio, Wuhan, China	GB23303	IHC
**Anti-GDF8 / Myostatin**	Abcam, Cambridge, UK	ab203076	WB
**Anti-BDNF**	Abcam, Cambridge, UK	ab108319	WB
**GAPDH**	Proteintech, Wuhan, China	10494-1-AP	WB

### Immunofluorescence (IF)

After paraffin sections were dewaxed, they were placed in citric acid antigen retrieval buffer (pH = 9.0) in a microwave oven for antigen retrieval. After the sections were blocked with serum, they were incubated with primary antibodies (see **Table 1** for reagent use) at 4°C overnight and then with secondary antibodies, after which the nuclei were counterstained with DAPI. The fluorescence images were captured with a Pannoramic 250 system (Pannoramic MIDI; 3D HISTECH, Budapest, Hungary), and then the images were viewed with CaseViewer (3D HISTECH) software. For quantification, the cross-sectional images were captured at 20x magnification in CaseViewer and digitized using Image J. After background correction, the mean OD and the fluorescence area of AChE, and the mean OD of SDH from the images were automatically detected in a double-blind manner. Statistical analysis was then performed. All quantization was performed using original, unedited images. Five sections per sample were imaged. Images were captured from n = 6 rats per group. For each experiment, data from all images for each rat were averaged and used for final statistical analysis.

### Western blot (WB) analysis

The soleus muscle and spinal cord samples were frozen in liquid nitrogen immediately after removal and stored at -80°C. Tissue homogenate protein extracts were used for WB detection (n = 3 per group). The expression of GDF8 protein in soleus muscle and the expression of BDNF (see **Table 1** for reagent use) protein in spinal cord was analyzed. Protein from the samples was separated by 10% SDS-PAGE and transferred to a PVDF membrane. The membrane was incubated with primary antibodies at 4°C overnight, after which the membrane was incubated with secondary antibodies (GAPDH) under shaking, developed in a dark room, and scanned after fixation. The band gray values were quantified by Image-Pro Plus and are expressed as ratios relative to the values for GAPDH.

### Statistical analysis

GraphPad Prism 8.0 (GraphPad Software, San Diego, California, USA) was used for statistical analysis. One-way ANOVA followed by Tukey’s multiple comparisons test was used to analyze differences among the Sham, SCI, Ex, Gs, and Ex+Gs groups. Two-way repeated measures ANOVA followed by Tukey’s multiple comparisons test were used to analyze differences in BBB scores among different groups. P < 0.05 represented statistical significance.

## Results

### GsMTx-4 combined with exercise significantly alleviated atrophic pathological changes in the soleus muscle after SCI

**Fig 2:**
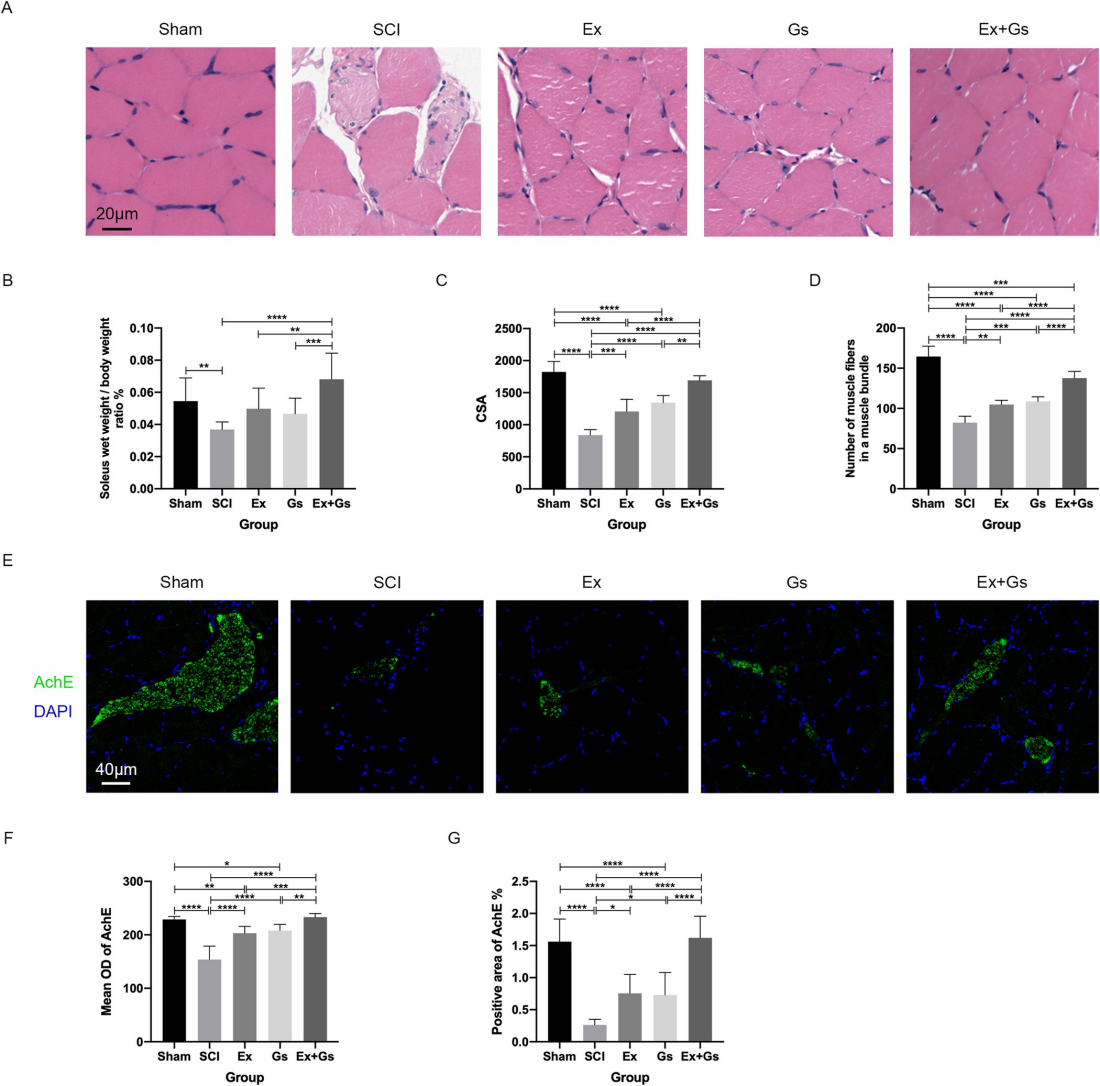
Combination therapy significantly ameliorated the atrophy of soleus muscles in rats after SCI. **(A)** The quantitative analysis of soleus muscle weight in rats. The soleus wet weight/ body weight ratio % was quantified. The data are presented as the means ± SDs, n = 6. One-way ANOVA followed by Tukey’s multiple comparisons test. **P < 0.01, ***P < 0.001, and ****P < 0.0001. **(B-D)** Representative images and the quantitative analysis of soleus muscle transverse sections stained with HE in rats. Scale bar = 20 μm. The CSA and number of muscle fibers in a muscle bundle were quantified. The data are presented as the means ± SDs, n = 6. One-way ANOVA followed by Tukey’s multiple comparisons test. **P < 0.01, ***P < 0.001, and ****P < 0.0001. **(E-G)** Representative images and the quantitative analysis of soleus muscle transverse sections stained with anti-AChE in rats. Scale bars = 40 μm. The mean OD of AChE and positive area of AChE% in the soleus muscle sections were quantified. The data are presented as the means ± SDs, n = 9. One-way ANOVA followed by Tukey’s multiple comparisons test. *P < 0.05, **P < 0.01, ***P < 0.001, and ****P < 0.0001. SCI: spinal cord injury.

The muscle wet weight ratio of the soleus muscle was calculated to evaluate the effect of combination therapy on soleus muscle atrophy in SCI rats (**Fig 2A**). Compared with the Sham group, the muscle wet weight ratio of the SCI group decreased significantly (P = 0.0075). Although the muscle wet weight ratio of the Ex group and Gs group showed an increasing trend, it was not statistically significant compared with the SCI group. Also, the muscle wet weight ratio of the Ex+Gs group was significantly increased compared with the SCI group (P<0.0001), while the muscle wet weight ratio of the Ex+Gs group was significantly higher than that of the Ex group and Gs group (P = 0.0049 and P = 0.0007, respectively), indicating that the decrease in wet weight of rat soleus muscle after SCI may be inhibited in the Ex+Gs group.

The number of muscle fibers and CSA of the soleus muscle were calculated to evaluate the effect of combination therapy on the structure of the soleus muscle in SCI rats (**Fig 2B–2D**). Compared with the Sham group, the soleus muscle CSA of the SCI, Ex, and Gs groups decreased significantly (all P<0.0001). In addition, compared with the SCI group, the soleus muscle CSA of the Ex group, Gs group, and Ex+Gs group was significantly increased (P = 0.0005, P<0.0001, and P<0.0001, respectively). Also, the soleus CSA of the Ex+Gs group was significantly higher than that of the Ex group and Gs group (P<0.0001 and P = 0.0010, respectively), indicating that all three interventions may contribute to an increase in soleus muscle CSA in SCI rats. Furthermore, compared with the Sham group, the number of soleus muscle fibers in the other four groups was significantly decreased (all P<0.0001). Compared with the SCI group, the number of muscle fibers in the Ex group, Gs group, and Ex+Gs group was significantly increased (P = 0.0010, P = 0.0001, and P<0.0001, respectively), indicating that all three interventions may contribute to increasing the number of muscle fibers in SCI rats. The number of muscle fibers in the Ex+Gs group was significantly higher than that in the Ex group and Gs group (both P<0.0001), indicating that among the three intervention groups, the Ex+Gs group had the most significant effect on the number of soleus muscle fibers in SCI rats.

In order to observe the functional status of the neuromuscular junction (NMJ), we performed AChE immunofluorescence staining on muscle tissue sections, after which we calculated the mean OD value of AChE and the proportion of AChE positive area within the visual field area (**Fig 2E–2G**). Compared with the Sham group, the AChE positive area % of the soleus muscle in the SCI group, Ex group, and Gs group was significantly reduced (all P<0.0001). Compared with the SCI group, the AChE positive area % of the soleus muscle in the Ex group, Gs group, and Ex+Gs group was significantly increased (P = 0.0110, P = 0.0173, and P<0.0001, respectively), indicating that all three intervention groups had positive effects on the AChE positive area% in the soleus muscle of SCI rats. The AChE positive area % of soleus muscle in the Ex+Gs group was significantly higher than that of the Ex group and Gs group (both P<0.0001), indicating that among the three intervention groups, the Ex+Gs group had the most significant positive effect on the AChE positive area% in the soleus muscle of SCI rats. Compared with the Sham group, the mean OD of AChE in the SCI, Ex, and Gs groups were significantly lower (P<0.0001, P = 0.0036, and P = 0.0265, respectively). In addition, compared with the SCI group, the mean OD of AChE in the Ex group, Gs group, and Ex+Gs group were all significantly increased (all P<0.0001), indicating that all three interventions had positive effects on the expression of AChE in the soleus muscle of SCI rats. The mean OD values of soleus muscle in the Ex+Gs group were significantly higher than those in the Ex group and Gs group (P = 0.0005 and P = 0.0047, respectively), indicating that among the three intervention groups, the Ex+Gs group had the most significant positive effect on the expression of AChE in the soleus muscle of SCI rats.

Overall, these data suggest that the Ex group, Gs group, and Ex+Gs group all had a certain effect on the structure of soleus muscle in SCI rats, with the Ex+Gs group having the most significant effect on inhibiting soleus muscle atrophy caused by SCI.

### GsMTx-4 combined with exercise significantly improved oxidative capacity and myostatin expression in the soleus muscle after SCI

**Fig 3:**
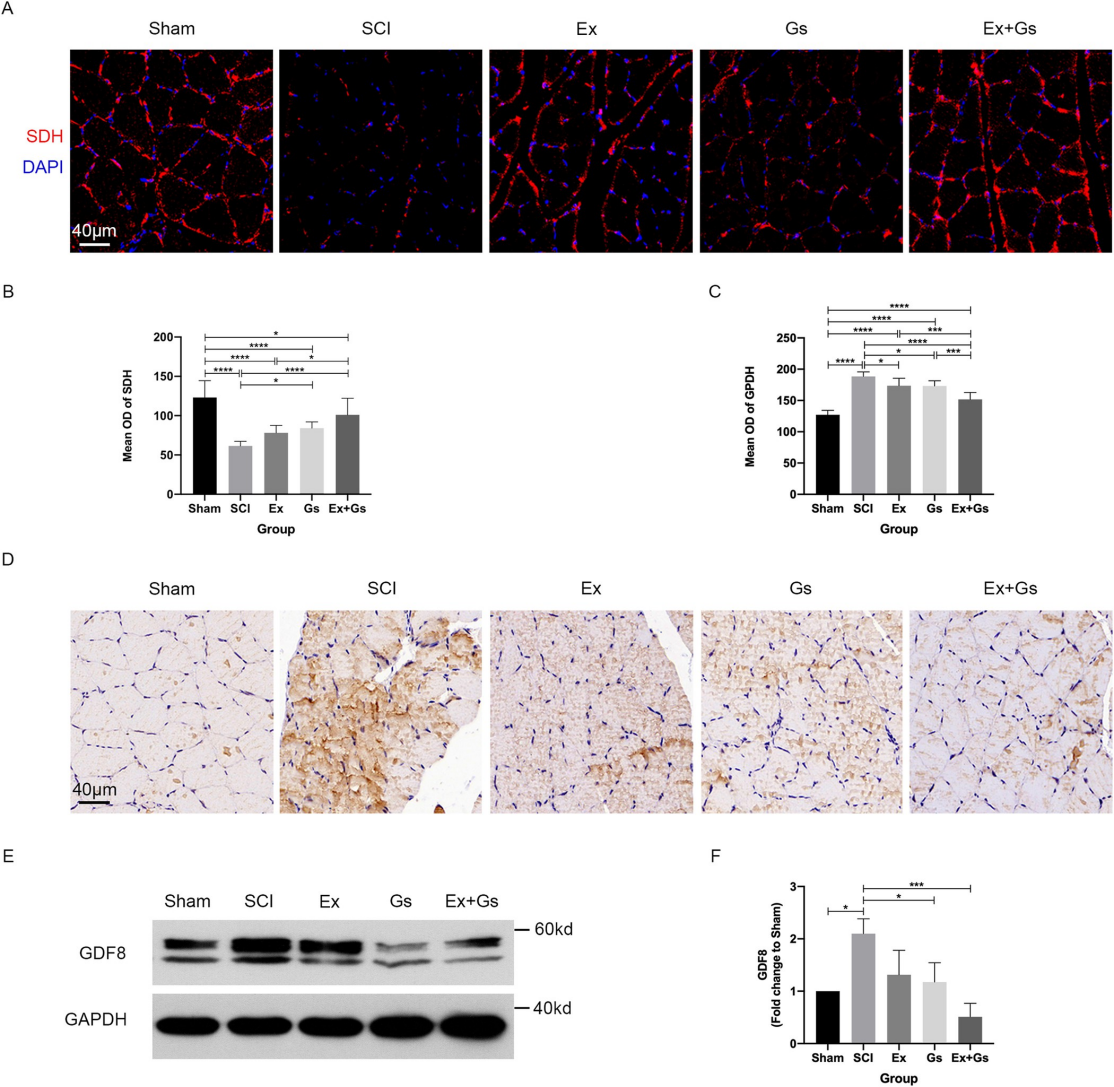
Combination therapy significantly improved oxidative capacity and myostatin expression in the soleus muscle after SCI. **(A** and **B)** Representative images and the quantitative analysis of soleus muscle transverse sections stained with anti-SDH in rats. Scale bars = 40 μm. The mean OD of SDH in the soleus muscle sections was quantified. The data are presented as the means ± SDs, n = 9. One-way ANOVA followed by Tukey’s multiple comparisons test. *P < .05 and ****P < .0001. **(C** and **D)** Representative images and the quantitative analysis of soleus muscle transverse sections stained with anti-GPDH in rats. Scale bars = 40 μm. The mean OD of GPDH in the soleus muscle sections was quantified. The data are presented as the means ± SDs, n = 9. One-way ANOVA followed by Tukey’s multiple comparisons test. *P < 0.05, ***P < 0.001, and ****P < 0.0001. **(E** and **F)** Changes in GDF8 expression in rats by WB. The soleus muscle tissue was fractionated to isolate lysosome-enriched fraction and then processed for Western blot. GAPDH was used as the loading control. The fold change relative to Sham was calculated. The data are presented as the means ± SDs, n = 3. One-way ANOVA followed by Tukey’s multiple comparisons test. *P < 0.05 and ***P < 0.001. SCI: spinal cord injury.

In order to explore the mechanism of changes in the morphological structure of rat soleus muscle, we subsequently examined the growth and metabolism of muscle cells. IF detected SDHA to reflect the aerobic oxidative capacity of muscle cells (**Fig 3A and 3B**). Compared with the Sham group, the mean OD of SDHA in the other four groups was significantly decreased (P<0.0001, P<0.0001, P<0.0001, and P = 0.0227, respectively). Compared with the SCI group, the mean OD of SDHA was significantly higher in both the Gs group and the Ex+Gs group (P = 0.0178 and P<0.0001, respectively), with the highest value observed in the Ex+Gs group, which suggests that the combination therapy may significantly enhance the aerobic oxidative capacity of soleus muscle in SCI rats. The mean OD of SDHA in the Ex+Gs group was significantly higher than in the Ex group (P = 0.0168).

We detected GPDH by IHC to reflect the glycolytic ability of muscle cells (**Fig 3C and 3D**). Compared with the Sham group, the mean OD of GPDH in the other four groups was significantly increased (all P<0.0001). Compared with the SCI group, the mean OD of GPDH in the Ex group, Gs group, and Ex+Gs group were significantly lower (P = 0.0141, P = 0.0108, and P<0.0001, respectively), indicating that all three intervention groups may have reduced the glycolytic ability of SCI rat soleus muscle. The mean OD of GPDH in the Ex+Gs group was significantly lower than in the Ex group and Gs group (P = 0.0001 and P = 0.0002, respectively), indicating that among the three intervention groups, combination therapy had the most significant effect on glycolysis in the soleus muscle of SCI rats.

Next, we detected the expression level of myostatin protein in muscle tissue by WB to reflect the growth and development of skeletal muscle (**Fig 3E and 3F**). Compared with the Sham group, the fold change of GDF8 was significantly higher after SCI (P = 0.0116), indicating increased expression of myostatin in muscle after SCI. Compared with the SCI group, there were no significant changes in the Ex group, the change folds of GDF8 in both the Gs group and the Ex+Gs group were significantly lower (P = 0.0328 and P = 0.0008, respectively), and the Ex+Gs group had the most significant statistical significance, indicating that the combination therapy has the most significant inhibitory effect on myostatin expression after SCI.

Overall, these data suggest that GsMTx-4 combined with exercise can significantly affect the oxidative and glycolytic abilities of the soleus muscle and inhibit the expression of myostatin protein in SCI rats.

### GsMTx-4 combined with exercise significantly alleviated pathological changes in the spinal cord after SCI

**Fig 4:**
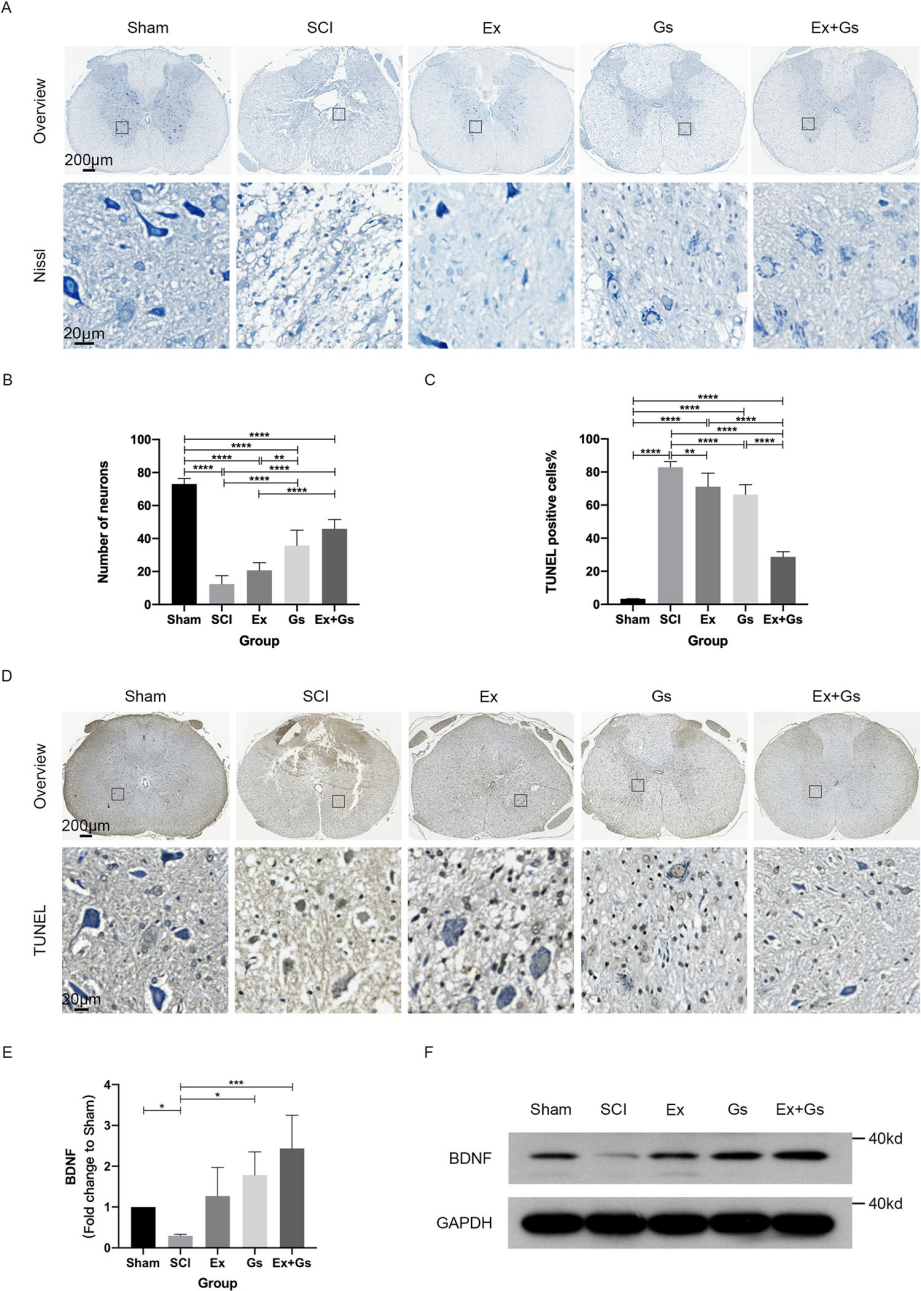
Combination therapy significantly alleviated neuronal loss in the spinal cord after SCI. **(A** and **B)** Representative images and the quantitative analysis of spinal cord transverse sections stained with Nissl in rats. Scale bar = 200 μm and 20 μm (overview and magnified image). The numbers of neurons in the ventral horn of the spinal cord were quantified. The data are presented as the means ± SDs, n = 6. One-way ANOVA followed by Tukey’s multiple comparisons test. **P < 0.01 and ****P < 0.0001. **(C and D)** Representative images of spinal cord transverse sections stained with TUNEL in rats. The red arrows indicate TUNEL-positive cells. Scale bar = 200 μm and 20 μm (overview and magnified image). The TUNEL-positive cells % in the spinal cord sections was quantified. The data are presented as the means ± SDs, n = 6. One-way ANOVA followed by Tukey’s multiple comparisons test. **P < 0.01 and ****P < 0.0001. SCI: spinal cord injury. **(E and F)** Changes in BDNF expression in rats by WB. The spinal cord tissue was fractionated to isolate lysosome-enriched fraction and then processed for WB. GAPDH was used as the loading control. The fold change relative to Sham was calculated. The data are presented as the means ± SDs, n = 4. One-way ANOVA followed by Tukey’s multiple comparisons test. *P < 0.05 and ***P < 0.001. SCI: spinal cord injury.

In order to explore the effects of different intervention methods on neurons in the spinal cord of SCI rats, the Nissl staining and TUNEL were used to observe the survival and apoptosis of motor neurons in the anterior horn of the spinal cord (**Fig 4A–4D**). Compared with the Sham group, the number of neurons in the SCI, Ex group, Gs group, and Ex+Gs group was significantly decreased (all P<0.0001). Compared with the SCI group, the number of neurons in the Gs and Ex+Gs groups significantly increased (both P<0.0001). Nevertheless, there were no significant changes in the Ex group compared to the SCI group, and the number of neurons in the Ex group was significantly lower than that in the Gs group and the Ex+Gs group (P = 0.0019 and P<0.001, respectively), indicating that the effect of exercise alone on the number of neurons in the anterior horn of the spinal cord after SCI was not significant. Combination therapy may have a higher protective effect on neurons than exercise alone.

The percentage of TUNEL-positive cells in the Ex group was significantly lower than that in the Gs group and Ex+Gs group (P = 0.0019 and P<0.0001, respectively). Compared with the Sham group, the percentage of TUNEL-positive cells in the other four groups was significantly increased (all P<0.0001). Compared with the SCI group, the percentage of TUNEL-positive cells in the Ex group, Gs group, and Ex+Gs group was significantly reduced (P = 0.0034, P<0.0001, and P<0.0001, respectively), indicating that all three interventions may reduce spinal cord cell apoptosis in SCI rats. The percentage of TUNEL-positive cells in the Ex+Gs group was significantly higher than in the Ex group and the Gs group (both P<0.0001), indicating that the combination therapy has the most significant inhibitory effect on spinal cord cell apoptosis in SCI rats.

In order to explore the causes of pathological changes in neurons in the spinal cord of SCI rats, WB was used to detected the expression level of BDNF protein (**Fig 4E and 4F**). Compared with the Sham group, the fold change of BDNF was significantly lower after SCI (P = 0.0498). Compared with the SCI group, the change folds of BDNF in both the Ex group, the Gs group and the Ex+Gs group were significantly higher (P = 0.0070, P<0.0001 and P<0.0001, respectively). Compared with the Ex group, the change folds of BDNF in the Ex+Gs group were significantly higher (P = 0.0033), indicating that the combination therapy has the most significant promotion effect on BDNF expression after SCI.

Overall, these data suggest that the combination therapy effectively improves the pathological changes in the spinal cord of SCI rats.

### GsMTx-4 combined with exercise significantly improved the motor function of rats after SCI

**Fig 5:**
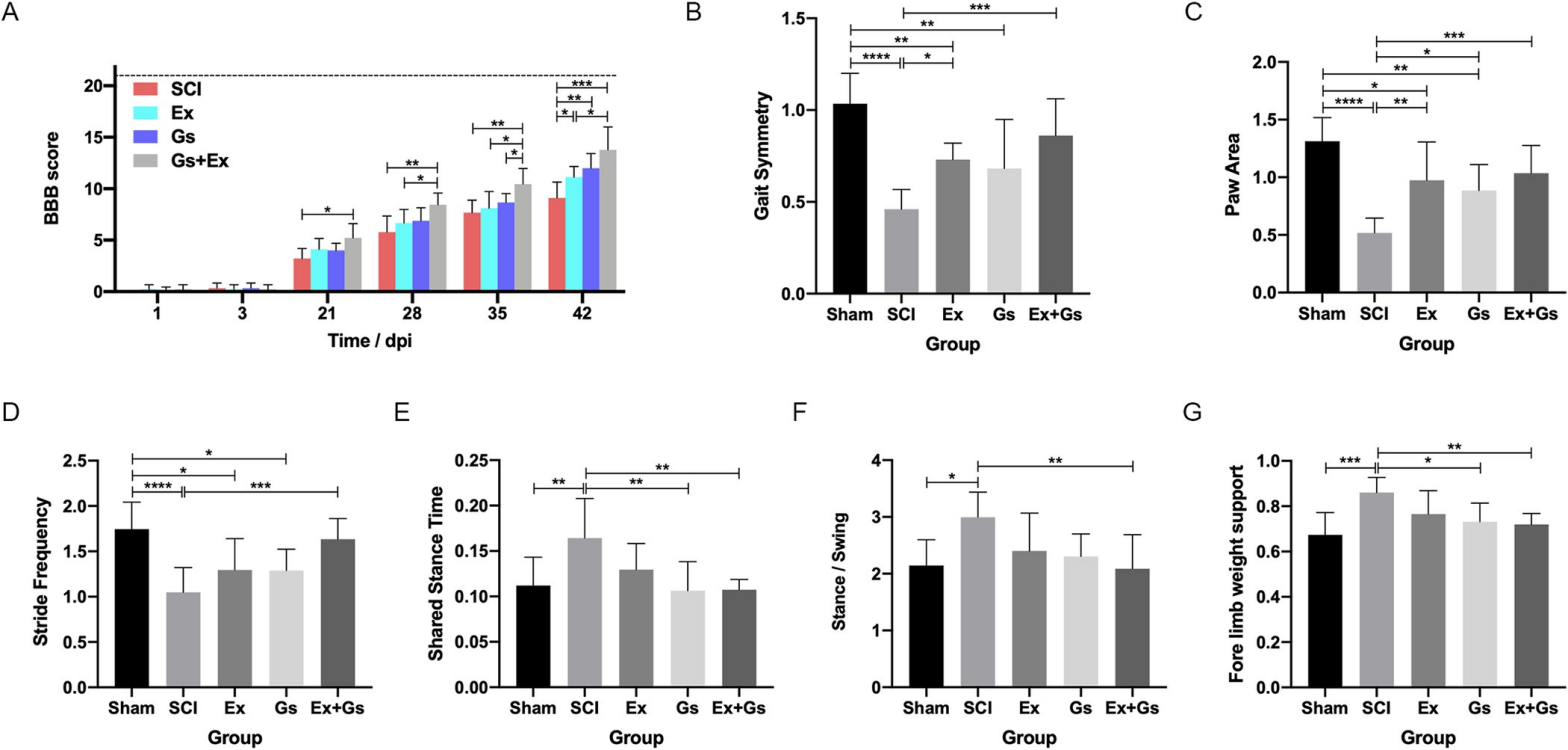
BBB scores and gait analysis. **(A)** BBB scores on day 1, 3, 21, 28, 35, and 42 after modeling in all groups. **(B-G)** The results of gait data analysis—gait symmetry, hind paw area, stride frequency, shared stance time, stance/swing, and forelimb weight support—for the Sham, SCI, Ex, Gs, and Ex+Gs groups at day 42 after modeling. The data are presented as the means ± SDs, n = 9. One-way ANOVA followed by Tukey’s multiple comparisons test. *P < 0.05, **P < 0.01, ***P < 0.001, and ****P < 0.0001. BBB: Basso-Beattie-Bresnahan; SCI: spinal cord injury.

The behavior performance was scored using the BBB, which was used to evaluate the effect of combination therapy on the motor function of rats (**Fig 5A**). The BBB scores of the Sham group were all 21 points. During the 4 weeks of exercise, the BBB scores of the Ex+Gs group were significantly higher than those of the SCI group (1–4 weeks: P = 0.0152; P = 0.0045; P = 0.0031; P = 0.0007, respectively), indicating that the BBB score of rats significantly improved within 1–4 weeks after combination therapy. At 2w after exercise, the BBB score of the Ex+Gs group was significantly higher than that of the Ex group (P = 0.0343). In the 3rd week, the BBB scores of the Ex+Gs group were significantly higher than those of the Ex group and Gs group (P = 0.0277 and P = 0.0402, respectively). Four weeks after exercise, the BBB scores of the Ex group and the Gs group were significantly higher than those of the SCI group (P = 0.0276 and 0.0038, respectively); however, the statistical significance was the most significant in the Ex+Gs group, indicating that the most significant improvement in BBB scores was observed in rats after combination therapy. Although no group returned to 21 points at the end of the experiment, the scores of the three intervention groups improved at all times compared with the Sham group, indicating that all three interventions may have somewhat improved the BBB score of SCI rats.

The gait of rats was carefully assessed using the Digi-gait gait analysis system (**Fig 5B–5G**). Compared with the Sham group, the stance/swing ratio of the SCI group was significantly increased (P = 0.0109). Compared with the SCI group, the stance/swing ratio of the Ex+Gs group was significantly reduced (P = 0.0059), indicating that during walking, the contact time of the paws with the treadmill (indicating weight support) was reduced, and the swing time of paws in the air was increased, which represents an improvement in gait. Although the stance/swing ratio of the Ex group and Gs group also showed a decreasing trend, it was not statistically significant (P = 0.1322 and P = 0.0572, respectively), indicating limited gait improvement. Compared with the Sham group, the stride frequency of the SCI, Ex, and Gs groups significantly decreased (P<0.0001, P = 0.012, and P = 0.0107, respectively). Also, compared with the SCI group, although the stride frequency of the Ex and Gs groups showed an increasing trend, there was no statistically significant difference. Compared with the SCI group, the stride frequency of the Ex+Gs group significantly increased (P = 0.0006). Compared with the SCI group, Ex and Gs groups were significantly reduced (P<0.0001, P = 0.0315, and P = 0.0037, respectively). Compared with the SCI group, the paw area of the Ex group, Gs group, and Ex+Gs group all increased significantly (P = 0.0017; P = 0.0156; P = 0.0003), and the paw area of the Ex+Gs group increased most significantly.

Compared with the Sham group, the hindlimb shared stance of rats in the SCI group was significantly increased (P = 0.0078), indicating that the animal posture was unstable. Compared with the SCI group, the hindlimb shared stance was significantly reduced in both the Gs group and the Ex+Gs group (P = 0.0027 and P = 0.0033, respectively), indicating that the alternating movement of the hind limbs was improved, meaning the posture of rats was more stable.

The gait symmetry of rats in the Sham group was close to 1 (1.034±0.1665), indicating better coordination between the forelimbs and hindlimbs. Compared with the Sham group, the gait symmetry in the SCI, Ex, and Gs groups was significantly reduced (P<0.0001, P = 0.0068, and P = 0.0013, respectively). Compared with the SCI group, the gait symmetry in the Ex group and Ex+Gs group was significantly increased (P = 0.0206 and P = 0.0002, respectively), indicating improved coordination between the forelimbs and hindlimbs.

Compared with the Sham group, the SCI group showed a significant increase in forelimb weight support (P = 0.0002). Compared with the SCI group, the forelimb weight support in the Gs group and Ex+Gs group was significantly reduced (P = 0.0159 and P = 0.0074, respectively). Overall, these data suggest that all three intervention groups had a specific positive impact on the gait, posture stability, and coordination of SCI rats, and the improvement was most significant after the combination therapy.

## Discussion

In this study, we explored the combined and individual efficacy of exercise training and GsMTx-4 on soleus muscle and motor function in rats with SCI. Compared with GsMTx-4/exercise alone, the combination therapy improved motor function, soleus muscle structure improvement, and neuron protection of the spinal cord in rats with SCI.

After SCI, skeletal muscle, the primary organ that produces movement, may undergo a process in which its mass could be reduced by 30–60%, depending on muscle type and severity of the lesion [35]. Studies have shown that the adaptive response to SCI occurs in slow-twitch muscle fibers more than fast-twitch muscle fibers and extensor muscle groups more than flexor muscle groups [36–38]. The soleus is a slow-twitch postural extensor muscle particularly susceptible to changes in weight-bearing status. Therefore, we mainly evaluated the wet weight ratio, muscle fiber CSA, and related protein expression of the soleus muscle. In this study, the muscle wet weight ratio and the muscle fiber CSA of rat soleus muscle were significantly reduced after SCI compared with normal rats, which is consistent with previous research [32]. Although exercise training or GsMTx-4 alone may not reverse the decrease in muscle wet-weight ratio after SCI and showed only a modest effect on restoring muscle CSA, the combination therapy significantly increased the wet-weight ratio and CSA in the soleus muscle, indicating that the combination therapy may contribute to inhibit muscle atrophy of soleus muscle in rats after SCI. The NMJ is a special structure that connects axon terminals between motor neurons and muscles [39]. As synapses, the integrity of NMJs is critical for transmitting motor neuron signals that initiate skeletal muscle contraction [40]. AChE is concentrated and retained at the neuromuscular junction and is used as a marker of neuromuscular junction development, reflecting the functional status of the NMJ [41]. In this study, we observed that the decrease in AChE expression caused by SCI was reversed after the combination therapy, indicating that the combination therapy exerts a protective effect on the integrity of the NMJ. An in vitro study found that GsMTx-4 could promote the desensitization of nicotinic acetylcholine receptors, preventing neurotoxicity caused by receptor overactivation and protecting the integrity of NMJ [42]. In various experimental models, Piezo1 has been found to play a significant role in promoting muscle hypertrophy and muscle fiber type transformation. Mechanical stretching applied to isolated mouse muscles to activate Piezo1 was shown to facilitate Ca^2+^ influx, promoting the fusion of muscle stem cells (MuSCs) into multinucleated cells or muscle fibers [43]. Additionally, cell-based experiments of mouse MuSCs demonstrated that Piezo1 knockout leads to a compensatory increase in Ca^2+^ influx through T-type voltage-gated Ca^2+^ channels, inducing NOX4 expression via the cPKC pathway, thereby elevating reactive oxygen species (ROS) levels, which trigger cell senescence and death [44]. Most current studies primarily focus on skeletal muscle itself rather than on models of neural injury. Future research is needed to further explore the role of GsMTx-4 in skeletal muscle changes after SCI.

The SDH activity is a marker of cellular aerobic oxidative capacity, while GPDH is a marker of cellular glycolysis [45]. Slow-twitch muscle fibers have the highest SDH activity and the lowest GPDH activity in skeletal muscle. The fatigue of skeletal muscle increases after SCI and is related to the activity of SDH [46]. In this study, SDH activity decreased, while GPDH activity increased in the soleus muscle of rats after SCI compared with the Sham group, which is consistent with previous research [47–50]. SDH activity in the soleus muscle increased after the combination therapy compared with the SCI group, indicating that the oxidative capacity of the soleus muscle increased after the combination therapy. The inhibition of muscle atrophy induced by the combination therapy may be partly due to increased oxidative capacity. In mouse models, activation of the mTOR signaling pathway has been shown to enhance SDH enzyme activity, promote the formation of slow-twitch muscle fibers, and improve mitochondrial function in skeletal muscle, thereby boosting endurance performance. These effects, however, can be reversed by mTOR inhibitors [51]. This suggests a potential correlation between skeletal muscle oxidative capacity and the mTOR pathway, warranting further exploration into its role within SCI models.

In order to explore the mechanism through which the combination therapy inhibits muscle atrophy of soleus muscle in rats after SCI, we detected changes in GDF8 protein levels. GDF-8, also known as myostatin, is highly expressed in skeletal muscle and is a muscle-derived negative regulator of muscle growth [52]. It inhibits muscle growth, development, and regeneration, and GDF-8 deficiency significantly increases muscle mass [53]. Increased myostatin gene expression was observed in both SCI patients and rodent SCI models [54, 55], which is consistent with the results of this study. Our results showed that the combination therapy reversed the increase in GDF-8 expression caused by SCI, indicating that the anti-muscle atrophy effect caused by the combination therapy may be partly due to the inhibition of the negative regulatory effect of GDF-8 on muscle growth. The growth effect of muscle fibers may improve motor function after SCI [56, 57]. It has been reported that dietary restriction in lambs suppresses mTOR expression in the semitendinosus muscle, while both mRNA and protein levels of GDF-8 are elevated [58]. This indicates that mTOR may influence skeletal muscle structure and function by regulating GDF-8. Moreover, both exercise and Piezo1 are closely related to the mTOR signaling pathway. In a rat model of cerebral ischemia-reperfusion injury, abnormal upregulation of Piezo1 was found to promote neuronal apoptosis and autophagy via the AMPK-mTOR signaling axis [59]. Exercise, on the other hand, activates the mTOR pathway and contributes to skeletal muscle mass gain [60]. Therefore, the mTOR pathway might be a potential mechanism through which the combination therapy enhanced skeletal muscle function following SCI, warranting further investigation.

Our research revealed that the combination therapy increased SDH activity, enhanced muscle oxidative capacity, and reduced the expression of GPDH and GDF-8. We propose that an interaction may exist between oxidative metabolism in muscle and the GDF-8 signaling pathway. Elevated SDH activity suggests improved mitochondrial oxidative phosphorylation, leading to increased ATP synthesis and enhanced cellular energy status [61]. Since SDH activity depends on adequate oxygen availability, the improved oxygen environment following the combination therapy might facilitate the full oxidation of pyruvate and efficient glucose utilization. This could reduce reliance on lipid metabolism, thereby decreasing the expression of GPDH [62]. Furthermore, studies have shown that GDF-8 inhibition significantly downregulates the Mss51 gene in mice, enhancing mitochondrial respiratory capacity and improving both oxygen consumption and metabolic efficiency of skeletal muscle fibers [63]. Further research is needed to determine whether the combination therapy modulates mitochondrial function, thereby influencing the expression of SDH, GPDH, and GDF-8.

Traumatic SCI is caused by mechanical forces, with the extent of damage varying based on the type of mechanical insult, such as penetrating injuries or tensile/compressive trauma [64, 65]. Under mechanical stimulation, Piezo1 may contribute to various pathological processes in SCI, including neuroinflammation, edema, neuronal death, and demyelination [22, 66–68]. Studies indicate that blocking Piezo1 channels with GsMTx-4 offers neuroprotective and regenerative benefits. For example, it has been shown to promote axonal growth in retinal ganglion cells [69], reduce hypersensitization of Piezo1 channels in astrocytes under neuroinflammatory conditions [70, 71], preserve neuronal survival after intracerebral hemorrhage [72], and mitigate autophagy overactivation caused by sleep deprivation to protect cognitive functions in the basal forebrain [73]. In our study, we observed that GsMTx-4 treatment during the acute phase of SCI may hold potential for promoting the repair of spinal cord neural structures. Although exercise training alone showed an improvement trend, it did not significantly reverse the death of motor neurons in the anterior horn of the spinal cord caused by SCI. GsMTx-4 treatment alone showed a modest protective effect on motor neurons in the anterior horn of the spinal cord. Combination therapy significantly increased the number of motor neurons in the anterior horn of the spinal cord and significantly reduced neuron apoptosis, indicating that combination therapy may effectively inhibit nerve damage after SCI. In the inner ear of zebrafish, Piezo1 has been observed to co-localize with BDNF protein, and their interaction may be associated with neuronal plasticity and regeneration [74]. Furthermore, in a rat model of sleep deprivation, activation of Piezo1 was found to mediate the cleavage of TrkB via the downstream calcium-dependent cysteine protease Calpain. This process inhibited BDNF-TrkB signaling and led to secondary neuronal damage, which could be reversed by the Piezo1 inhibitor GsMTx-4 [75]. Similar phenomena have also been observed in mice [73]. In this study, exercise training may effectively improve the neuroprotective and repairing effects on spinal cord tissue caused by GsMTx-4, which may be related to promoting BDNF secretion. Future studies should delve deeper into the mechanisms underlying the interaction between Piezo1 inhibition and neurotrophic factors such as BDNF.

BWSTT can potentially promote neuroplasticity after mild to moderate motor incomplete SCI and improve walking speed, temporal gait parameters, and lower limb muscle activation patterns [76–78]. Structural and functional plasticity may drive motor recovery resulting from BWSTT and stems from the reorganization of both supraspinal and spinal cord neural circuits [79, 80]. In this study, the BBB score revealed that SCI rats showed spontaneous recovery of motor function according to a fixed pattern, which is similar to the results of previous studies [81, 82]. However, compared with the SCI group, the BBB scores of the three intervention groups significantly improved 42 days after injury, and the improvement effect of the combination therapy group was the most obvious. In addition, the results of the Digi-Gait analysis showed that compared with the SCI group, animals in the combination therapy group had better performance in stance/swing ratio, stride frequency, paw area, hindlimb shared stance, gait symmetry, and forelimb weight support. The improvement of these indicators indicates that the duration of contact between the hind paws and the treadmill is increased, the weight loading of the hind limbs is increased, the gait is more stable, and the movement control level of animals is higher [83].

The present study has some limitations. As we only observed the status of spinal cord neurons, future systematic studies on the effects of combination therapy on the entire functional network are needed. At the same time, the therapeutic effects of different exercise intensities may differ, so different exercise programs need to be explored in more detail. This study utilized a single dose and administration method of GsMTx-4. Exploring different dosing regimens or sustained-release formulations in the future would provide more comprehensive insights. Further investigations into the underlying mechanisms are essential to ensure the safety and efficacy of GsMTx-4 in primate models and clinical trials, facilitating its practical application and broader adoption. Additionally, combined therapeutic strategies should be further refined and optimized to maximize their therapeutic potential.

## Conclusions

The results of this study revealed that GsMTx-4 combined with exercise has a better effect on the histopathology and motor function of rats with SCI compared with GsMTx-4/exercise alone. The combination therapy potentially reversed the structural atrophic changes in the soleus muscle caused by SCI, showing a significant protective effect on motor neurons of the spinal cord anterior horn, which may be related to the recovery of motor function in rats.

## Supporting information

S1 Raw images Raw image data of western blots. (PDF)

S1 Table Raw data of Fig 2. (DOCX)

S2 Table Raw data of Fig 3. (DOCX)

S3 Table Raw data of Fig 4. (DOCX)

S4 Table Raw data of Fig 5. (DOCX)
