# Supplementary figures and images for: GsMTx-4 combined with exercise improves skeletal muscle structure and motor function in rats with spinal cord injury
Source: PLoS One. 2025 Jan 22;20(1):e0317683. doi: 10.1371/journal.pone.0317683 (PMC11753701; doi:10.1371/journal.pone.0317683)

Raw image of western blots in Fig 3E

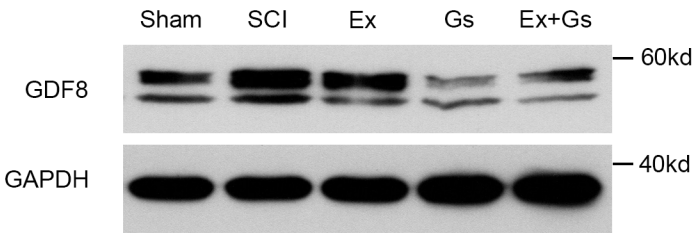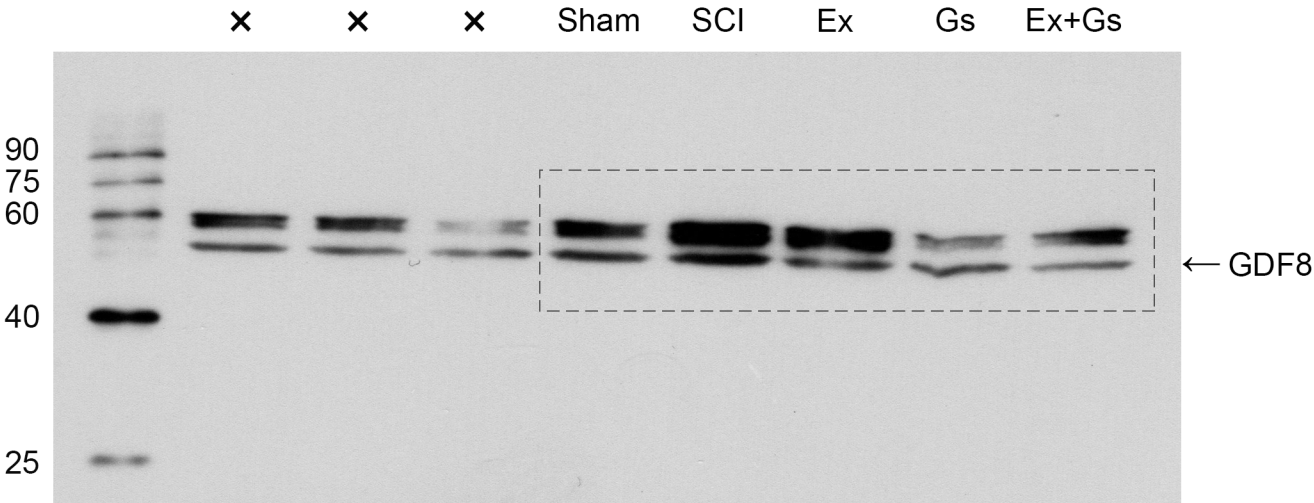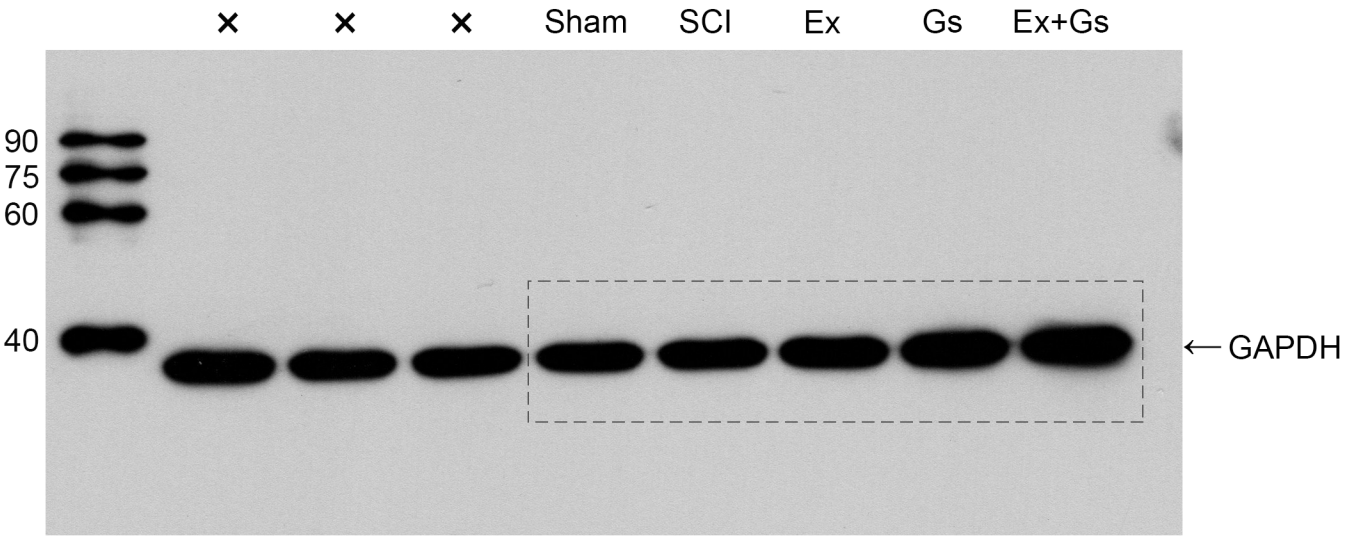

Raw image of western blots in Fig 4F

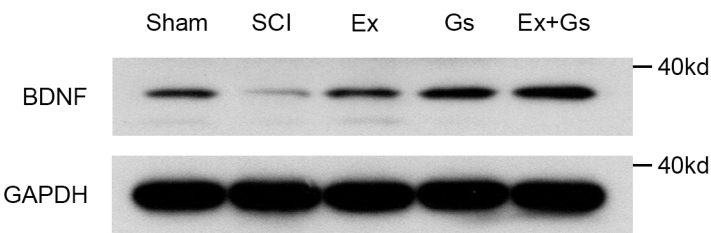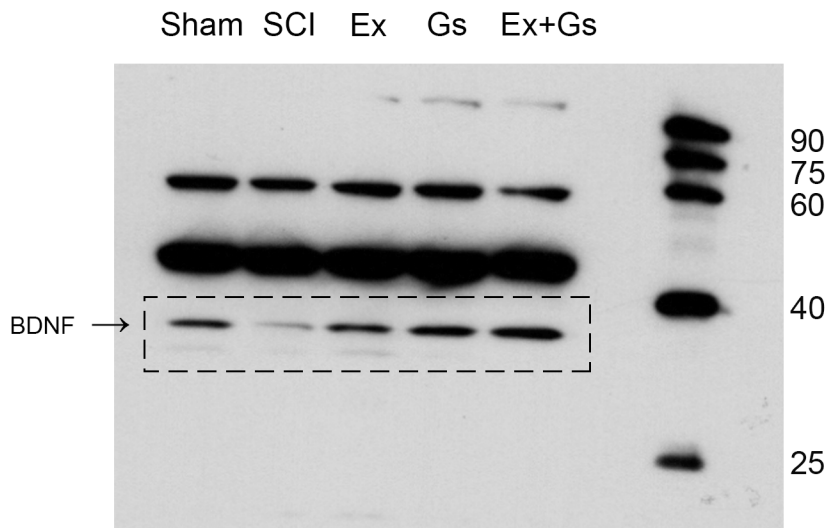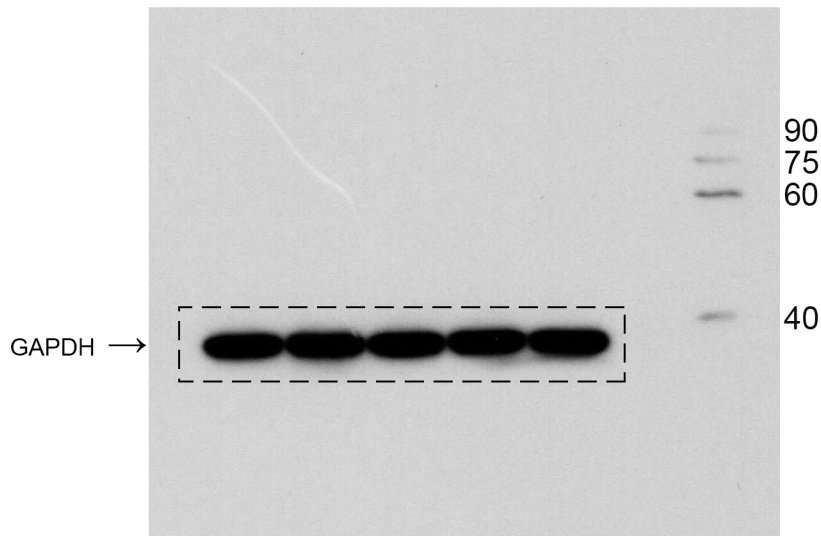

Supplement: S1 Raw images — (PDF) [file pone.0317683.s001.pdf]
