# Supplementary material for: GsMTx-4 combined with exercise improves skeletal muscle structure and motor function in rats with spinal cord injury
Source: PLoS One. 2025 Jan 22;20(1):e0317683. doi: 10.1371/journal.pone.0317683 (PMC11753701; doi:10.1371/journal.pone.0317683)
Supplement: S1 Table — (DOCX) [file pone.0317683.s002.docx]

**Supplementary Table 1. The raw data of Fig 2**

|  | **Sham** | **SCI** | **Ex** | **Gs** | **Ex+Gs** |
| --- | --- | --- | --- | --- | --- |
| **soleus wet**  **weight /**  **bodyweight**  **ratio %**  **(both sides)** | 0.0475 | 0.0401 | 0.0434 | 0.0399 | 0.0670 |
|  | 0.0573 | 0.0397 | 0.0588 | 0.0573 | 0.0480 |
|  | 0.0526 | 0.0323 | 0.0559 | 0.0541 | 0.0646 |
|  | 0.0512 | 0.0391 | 0.0480 | 0.0318 | 0.0589 |
|  | 0.0437 | 0.0299 | 0.0539 | 0.0440 | 0.0522 |
|  | 0.0420 | 0.0318 | 0.0491 | 0.0574 | 0.0416 |
|  | 0.0918 | 0.0401 | 0.0515 | 0.0287 | 0.0805 |
|  | 0.0433 | 0.0458 | 0.0347 | 0.0514 | 0.0646 |
|  | 0.0408 | 0.0370 | 0.0524 | 0.0514 | 0.0850 |
|  | 0.0588 | 0.0348 | 0.0768 | 0.0523 | 0.0897 |
|  | 0.0695 | 0.0393 | 0.0237 | 0.0520 | 0.0757 |
|  | 0.0561 | 0.0329 | 0.0490 | 0.0395 | 0.0899 |
| **mean ± SD** | 0.0546±0.0144 | 0.0369±0.0046 | 0.0498±0.0129 | 0.0467±0.0097 | 0.0681±0.0163 |
| **CSA** | 1900.79 | 815.18 | 1042.52 | 1252.29 | 1768.04 |
|  | 2105.31 | 815.23 | 1094.74 | 1318.46 | 1632.92 |
|  | 1832.92 | 974.29 | 1099.93 | 1355.93 | 1598.57 |
|  | 1698.14 | 711.27 | 1319.90 | 1214.46 | 1749.02 |
|  | 1723.62 | 840.25 | 1542.01 | 1515.19 | 1748.41 |
|  | 1681.26 | 874.25 | 1149.30 | 1413.31 | 1654.46 |
| **mean ± SD** | 1823.67±162.11 | 838.41±86.01 | 1208.07±189.43 | 1344.94±109.63 | 1691.90±71.89 |
| **Number of muscle fibers** | 163.2 | 81.8 | 106.0 | 107.8 | 146.4 |
|  | 156.0 | 78.2 | 106.2 | 102.1 | 145.8 |
|  | 154.8 | 75.4 | 113.8 | 118.3 | 141.8 |
|  | 160.0 | 93.4 | 98.2 | 112.3 | 125.8 |
|  | 163.2 | 74.3 | 102.0 | 105.8 | 136.0 |
|  | 190.0 | 90.7 | 102.6 | 105.8 | 130.2 |
| **mean ± SD** | 164.53±12.96 | 82.30±8.03 | 104.80±5.30 | 108.68±5.78 | 137.67±8.47 |
| **Mean OD of AchE** | 237.25 | 125.62 | 213.38 | 210.46 | 238.43 |
|  | 221.79 | 132.91 | 219.12 | 195.22 | 227.76 |
|  | 237.77 | 141.06 | 203.44 | 194.63 | 234.94 |
|  | 226.26 | 124.46 | 189.46 | 223.69 | 238.06 |
|  | 225.52 | 145.93 | 192.82 | 199.58 | 238.23 |
|  | 225.26 | 186.86 | 189.79 | 213.40 | 240.88 |
|  | 229.63 | 164.85 | 206.63 | 225.22 | 224.73 |
|  | 227.31 | 183.03 | 221.18 | 209.39 | 222.27 |
|  | 228.90 | 179.63 | 193.35 | 201.82 | 232.84 |
| **mean ± SD** | 228.85±5.40 | 153.82±25.13 | 203.24±12.58 | 208.16±11.35 | 233.13±6.70 |
| **Positive area of AchE%** | 1.93 | 0.17 | 0.48 | 0.96 | 2.01 |
|  | 2.07 | 0.14 | 0.45 | 0.82 | 1.53 |
|  | 1.58 | 0.26 | 0.67 | 0.91 | 1.92 |
|  | 1.60 | 0.31 | 1.02 | 0.22 | 1.03 |
|  | 0.99 | 0.19 | 1.33 | 1.37 | 1.23 |
|  | 1.60 | 0.36 | 0.76 | 0.33 | 1.91 |
|  | 1.67 | 0.29 | 0.67 | 0.79 | 1.52 |
|  | 1.56 | 0.40 | 0.95 | 0.51 | 1.87 |
|  | 1.06 | 0.26 | 0.47 | 0.67 | 1.55 |
| **mean ± SD** | 1.56±0.35 | 0.26±0.09 | 0.76±0.30 | 0.73±0.35 | 1.62±0.34 |
