# Supplementary material for: GsMTx-4 combined with exercise improves skeletal muscle structure and motor function in rats with spinal cord injury
Source: PLoS One. 2025 Jan 22;20(1):e0317683. doi: 10.1371/journal.pone.0317683 (PMC11753701; doi:10.1371/journal.pone.0317683)
Supplement: S2 Table — (DOCX) [file pone.0317683.s003.docx]

**Supplementary Table 2. The raw data for Mean OD of SDH in Fig 3**

|  | Sham | SCI | Ex | Gs | Ex+Gs |
| --- | --- | --- | --- | --- | --- |
| 1 | 141.08 | 64.80 | 81.36 | 89.05 | 154.59 |
| 2 | 149.96 | 59.21 | 79.72 | 86.12 | 99.16 |
| 3 | 103.07 | 59.57 | 79.40 | 78.17 | 105.97 |
| 4 | 146.14 | 68.41 | 77.04 | 86.87 | 82.68 |
| 5 | 105.97 | 67.55 | 72.41 | 88.07 | 92.43 |
| 6 | 102.00 | 61.17 | 95.63 | 85.22 | 93.05 |
| 7 | 102.00 | 65.22 | 60.04 | 79.30 | 91.40 |
| 8 | 144.04 | 57.79 | 75.77 | 68.96 | 94.15 |
| 9 | 113.73 | 49.96 | 82.23 | 96.44 | 96.06 |
| mean ± SD | 123.11±21.47 | 61.52±5.76 | 78.18±9.38 | 84.24±7.85 | 101.05±21.02 |

**Supplementary Table 3. The raw data for Mean OD of GPDH in Fig 3**

|  | Sham | SCI | Ex | Gs | Ex+Gs |
| --- | --- | --- | --- | --- | --- |
| 1 | 138.25 | 180.46 | 186.99 | 173.42 | 133.70 |
| 2 | 131.02 | 179.40 | 158.71 | 173.23 | 144.08 |
| 3 | 132.64 | 198.67 | 167.33 | 179.43 | 156.29 |
| 4 | 123.17 | 194.34 | 172.90 | 167.49 | 149.32 |
| 5 | 127.00 | 188.69 | 180.46 | 175.57 | 155.94 |
| 6 | 125.42 | 190.15 | 179.40 | 177.67 | 168.14 |
| 7 | 114.52 | 195.34 | 173.42 | 165.45 | 166.59 |
| 8 | 120.43 | 179.36 | 154.26 | 187.24 | 146.50 |
| 9 | 131.74 | 189.28 | 188.69 | 158.81 | 144.84 |
| mean ± SD | 127.13±7.19 | 188.41±7.23 | 173.57±11.85 | 173.15±8.38 | 151.71±11.14 |

**Supplementary Table 4. The raw data for GDF8 in Fig 3**

|  | Sham | SCI | Ex | Gs | Ex+Gs |
| --- | --- | --- | --- | --- | --- |
| 1 | 1.00 | 2.19 | 1.85 | 1.20 | 0.31 |
| 2 | 1.00 | 2.33 | 1.06 | 0.80 | 0.42 |
| 3 | 1.00 | 1.78 | 1.03 | 1.53 | 0.80 |
| mean ± SD | 1.00±0.00 | 2.10±0.29 | 1.31±0.47 | 1.18±0.37 | 0.51±0.26 |
