# Supplementary material for: GsMTx-4 combined with exercise improves skeletal muscle structure and motor function in rats with spinal cord injury
Source: PLoS One. 2025 Jan 22;20(1):e0317683. doi: 10.1371/journal.pone.0317683 (PMC11753701; doi:10.1371/journal.pone.0317683)
Supplement: S3 Table — (DOCX) [file pone.0317683.s004.docx]

**Supplementary Table 5. The raw data for number of neurons in Fig 4**

|  | Sham | SCI | Ex | Gs | Ex+Gs |
| --- | --- | --- | --- | --- | --- |
| 1 | 78 | 20 | 24 | 54 | 44 |
| 2 | 72 | 9 | 28 | 29 | 48 |
| 3 | 70 | 8 | 20 | 32 | 40 |
| 4 | 69 | 12 | 17 | 35 | 48 |
| 5 | 73 | 17 | 15 | 29 | 55 |
| 6 | 76 | 8 | 20 | 35 | 40 |
| mean ± SD | 73.00±3.46 | 12.33±5.09 | 20.67±4.72 | 35.67±9.37 | 45.83±5.74 |

**Supplementary Table 6. The raw data for TUNEL-positive cells % in Fig 4**

|  | Sham | SCI | Ex | Gs | Ex+Gs |
| --- | --- | --- | --- | --- | --- |
| 1 | 3.41 | 85.37 | 74.00 | 76.47 | 26.23 |
| 2 | 3.30 | 79.49 | 73.68 | 65.00 | 25.76 |
| 3 | 3.16 | 82.61 | 79.07 | 64.52 | 29.03 |
| 4 | 3.53 | 80.56 | 61.29 | 60.71 | 30.16 |
| 5 | 3.45 | 88.46 | 60.61 | 61.54 | 33.87 |
| 6 | 3.26 | 80.56 | 78.13 | 70.21 | 27.37 |
| mean ± SD | 3.35±0.14 | 82.84±3.46 | 71.13±8.18 | 66.41±5.96 | 28.74±3.02 |

**Supplementary Table 7. The raw data for** **BDNF in Fig 4**

|  | Sham | SCI | Ex | Gs | Ex+Gs |
| --- | --- | --- | --- | --- | --- |
| 1 | 1 | 0.25 | 0.68 | 2.11 | 2.35 |
| 2 | 1 | 0.33 | 1.72 | 1.52 | 2.3 |
| 3 | 1 | 0.31 | 1.48 | 1.45 | 1.75 |
| 4 | 1 | 0.32 | 1.04 | 2.27 | 2.58 |
| mean ± SD | 1.00±0.00 | 0.30±0.04 | 1.23±0.46 | 1.84±0.41 | 2.25±0.35 |
