# Supplementary material for: GsMTx-4 combined with exercise improves skeletal muscle structure and motor function in rats with spinal cord injury
Source: PLoS One. 2025 Jan 22;20(1):e0317683. doi: 10.1371/journal.pone.0317683 (PMC11753701; doi:10.1371/journal.pone.0317683)
Supplement: S4 Table — (DOCX) [file pone.0317683.s005.docx]

**Supplementary Table 8. The raw data of BBB score shown in Fig 5A (mean ± SD)**

|  | Sham | SCI | Ex | Gs | Ex+Gs |
| --- | --- | --- | --- | --- | --- |
| 1 dpi | 21.00±0.00 | 0.00±0.00 | 0.22±0.44 | 0.11±0.33 | 0.22±0.44 |
| 3 dpi | 21.00±0.00 | 0.33±0.50 | 0.22±0.44 | 0.33±0.50 | 0.22±0.44 |
| 21 dpi | 21.00±0.00 | 3.22±0.97 | 4.11±1.05 | 4.00±0.71 | 5.22±1.39 |
| 28 dpi | 21.00±0.00 | 5.78±1.56 | 6.67±1.32 | 6.89±1.27 | 8.44±1.13 |
| 35 dpi | 21.00±0.00 | 7.67±1.22 | 8.11±1.62 | 8.67±0.87 | 10.44±1.51 |
| 42 dpi | 21.00±0.00 | 9.11±1.54 | 11.11±1.05 | 12.00±1.41 | 13.78±2.22 |

**Supplementary Table 9. The raw data shown in Fig 5B-G**

|  | Sham | SCI | Ex | Gs | Ex+Gs |
| --- | --- | --- | --- | --- | --- |
| gait symmetry | 0.96 | 0.37 | 0.85 | 0.60 | 1.19 |
|  | 1.04 | 0.54 | 0.63 | 0.28 | 0.72 |
|  | 0.77 | 0.47 | 0.70 | 0.73 | 0.99 |
|  | 1.06 | 0.38 | 0.63 | 0.61 | 1.00 |
|  | 1.01 | 0.59 | 0.80 | 0.71 | 0.81 |
|  | 0.95 | 0.54 | 0.69 | 1.07 | 0.53 |
|  | 0.97 | 0.42 | 0.79 | 1.07 | 0.67 |
|  | 1.18 | 0.27 | 0.64 | 0.69 | 0.89 |
|  | 1.37 | 0.56 | 0.84 | 0.38 | 0.95 |
| mean ± SD | 1.03±0.17 | 0.46±0.11 | 0.73±0.09 | 0.68±0.27 | 0.86±0.20 |
| hind paw area | 1.23 | 0.36 | 0.41 | 1.09 | 1.59 |
|  | 1.58 | 0.39 | 0.83 | 0.74 | 1.06 |
|  | 1.41 | 0.39 | 0.75 | 1.09 | 1.19 |
|  | 1.06 | 0.51 | 0.67 | 0.68 | 0.93 |
|  | 1.49 | 0.48 | 1.24 | 1.31 | 0.81 |
|  | 1.12 | 0.62 | 1.39 | 0.71 | 0.95 |
|  | 1.40 | 0.69 | 1.08 | 0.68 | 1.04 |
|  | 1.04 | 0.53 | 1.08 | 0.87 | 0.96 |
|  | 1.51 | 0.70 | 1.34 | 0.81 | 0.81 |
| mean ± SD | 1.31±0.21 | 0.52±0.13 | 0.97±0.33 | 0.89±0.23 | 1.04±0.24 |
| stride frequency | 1.70 | 1.60 | 1.15 | 1.25 | 1.90 |
|  | 1.45 | 1.20 | 1.45 | 1.40 | 1.80 |
|  | 1.60 | 1.05 | 1.55 | 1.40 | 1.90 |
|  | 1.90 | 0.75 | 1.75 | 1.65 | 1.65 |
|  | 2.25 | 0.85 | 0.75 | 1.45 | 1.65 |
|  | 1.50 | 0.85 | 0.90 | 0.90 | 1.55 |
|  | 1.35 | 1.30 | 1.25 | 1.40 | 1.65 |
|  | 2.00 | 0.90 | 1.70 | 1.05 | 1.20 |
|  | 1.95 | 0.95 | 1.15 | 1.10 | 1.40 |
| mean ± SD | 1.74±0.30 | 1.05±0.27 | 1.29±0.35 | 1.29±0.23 | 1.63±0.23 |
| shared stance time | 0.08 | 0.17 | 0.11 | 0.11 | 0.11 |
|  | 0.15 | 0.15 | 0.13 | 0.09 | 0.13 |
|  | 0.09 | 0.12 | 0.09 | 0.09 | 0.12 |
|  | 0.07 | 0.20 | 0.10 | 0.08 | 0.10 |
|  | 0.10 | 0.16 | 0.17 | 0.06 | 0.10 |
|  | 0.11 | 0.10 | 0.16 | 0.11 | 0.09 |
|  | 0.14 | 0.25 | 0.13 | 0.12 | 0.10 |
|  | 0.15 | 0.15 | 0.12 | 0.15 | 0.11 |
|  | 0.13 | 0.17 | 0.16 | 0.16 | 0.11 |
| mean ± SD | 0.11±0.03 | 0.16±0.04 | 0.13±0.03 | 0.11±0.03 | 0.11±0.01 |
| stance/swing | 2.00 | 2.90 | 2.15 | 2.25 | 1.75 |
|  | 1.65 | 3.10 | 2.50 | 2.15 | 1.15 |
|  | 2.60 | 2.80 | 1.60 | 2.15 | 1.60 |
|  | 2.15 | 2.75 | 2.85 | 1.65 | 2.05 |
|  | 3.00 | 2.95 | 1.80 | 2.05 | 1.70 |
|  | 1.50 | 3.05 | 3.75 | 3.05 | 2.20 |
|  | 2.25 | 2.20 | 2.85 | 2.30 | 2.80 |
|  | 2.05 | 3.80 | 2.20 | 2.60 | 2.85 |
|  | 2.10 | 3.40 | 1.90 | 2.55 | 2.70 |
| mean ± SD | 2.14±0.45 | 2.99±0.44 | 2.40±0.67 | 2.31±0.40 | 2.09±0.60 |
| forelimb weight support | 0.54 | 0.82 | 0.82 | 0.81 | 0.71 |
|  | 0.60 | 0.89 | 0.79 | 0.83 | 0.73 |
|  | 0.62 | 0.83 | 0.96 | 0.62 | 0.63 |
|  | 0.54 | 0.90 | 0.60 | 0.75 | 0.75 |
|  | 0.79 | 0.71 | 0.77 | 0.68 | 0.73 |
|  | 0.76 | 0.92 | 0.64 | 0.75 | 0.70 |
|  | 0.75 | 0.92 | 0.79 | 0.81 | 0.81 |
|  | 0.72 | 0.90 | 0.77 | 0.73 | 0.70 |
|  | 0.74 | 0.85 | 0.75 | 0.60 | 0.72 |
| mean ± SD | 0.67±0.10 | 0.86±0.07 | 0.77±0.10 | 0.73±0.08 | 0.72±0.05 |
